# *Plumbago Zeylanica* (*Chitrak*) leaf image dataset: A comprehensive collection for botanical studies, herbal medicine research, and environmental analyses

**DOI:** 10.1016/j.dib.2023.109929

**Published:** 2023-12-09

**Authors:** Kailas Patil, Yogesh Suryawanshi, Amit Dhoka, Prawit Chumchu

**Affiliations:** a Vishwakarma University, Pune, India; b Kasetsart University, Sriracha, Thailand

**Keywords:** Classification, Image-based analysis, Leaf images, Plant health, Machine learning

## Abstract

The *Plumbago Zeylanica* (*Chitrak*) Leaf Image Dataset is a valuable resource for botanical studies, herbal medicine research, and environmental analyses. Comprising a total of 10,660 high-resolution leaf images, the dataset is meticulously categorized into three distinct classes: Unhealthy leaves (3343 images), Healthy leaves (5288 images), and Dried leaves (2029 images). These images were captured from the medicinal plant *Chitrak*, a species of paramount importance in traditional medicine and environmental contexts. Researchers and practitioners can benefit from this dataset's richness in terms of both quantity and quality, using it to develop and test algorithms for leaf classification and health assessment. The *Chitrak* leaf image dataset holds the potential to foster innovative investigations and applications within the domains of botany, medicine, and environmental sciences.

Specifications Table

**Table:** 

Subject	Applied Machine Learning, Agriculture
Specific subject area	Agronomy & Crop Science
Data format	Raw
Type of data	Image
Data collection	The dataset is collected at Vishwakarma University. The dataset consists of 10,660 images categorized into “Healthy,” “Unhealthy,” and “Dried” categories. These categories contain both “Single Images” and “Multiple Images,” and within the “Single Images” category, there are further divisions based on the viewpoint, including “Front Images,” “Back Images.” The leaves were photographed on various backgrounds to amplify the characteristics of the leaf in all types of background and lighting conditions. These photos were saved in jpg format and then later resized to 1024 × 768 pixels and 256 × 256 pixels to reduce the size of the dataset and preserve the important details
Data source location	Vishwakarma University, Kondhwa Budruk, Maharashtra, Pune, India Latitude: 18.4605° N Longitude: 73.8837° E
Data accessibility	Repository name: Plumbago Zeylanica (Chitrak) Leaf Image Dataset Data identification number: 10.17632/twpv7hhmgb.2 Direct URL to data: https://data.mendeley.com/datasets/twpv7hhmgb/2

## 1 Value of the Data


 

• This dataset comprises a substantial collection of 10,660 high-resolution leaf images, making it a comprehensive resource for researchers in various fields. The dataset size greatly enhances the potential for diverse analyses and model development.
• Data scientists and machine learning practitioners can leverage the dataset's size to develop and test algorithms for leaf classification and disease detection, benefitting from a vast and diverse dataset for robust model development.
• This dataset plays a crucial role in agricultural and environmental research, facilitating the creation of tools for monitoring plant health, detecting diseases early, and promoting sustainable agricultural practices.
• The dataset is an educational asset, facilitating hands-on learning in image analysis, classification techniques, and data science methods, enhancing students' comprehension of plant biology and machine learning.

## 2 Background

*Plumbago Zeylanica*, commonly known as *Chitrak*, stands as a botanical treasure with profound significance in traditional medicine and environmental studies. The plant contains bioactive compounds, such as plumbagin, that exhibit strong anti-inflammatory effects. These properties make *Chitrak* a valuable component in traditional medicine systems, where it is often used to alleviate pain, reduce inflammation, and promote overall well-being. Its medicinal properties have been harnessed for centuries, making it a focal point for researchers and practitioners in the realms of botany, medicine, and environmental sciences [9].

The creation of the *Chitrak* Leaf Image Dataset was motivated by the inherent medicinal properties and economic importance of the Chitrak plant. With its various medicinal applications and significant market value, industries demand high-quality Chitrak plant parts, including roots, leaves, and flowers. The health and quality of these plant components are crucial for the pharmaceutical and herbal medicine industries. To address this need, we designed the dataset to empower machine learning models to discern and assess the quality of Chitrak leaves specifically. This not only serves the interests of industries seeking premium medicinal plant materials but also benefits farmers by providing a tool for quality evaluation in their cultivation practices. By leveraging machine learning algorithms to analyze leaf characteristics, we aim to contribute to the optimization of Chitrak cultivation, ensuring that the plant meets the stringent quality standards required by various stakeholders in the herbal and pharmaceutical sectors. In order to propel these investigations further, we present the *Chitrak* Leaf Image Dataset, a comprehensive repository tailored to enrich and advance these multidisciplinary fields.

## 3 Data Description

**Fig. 1:**
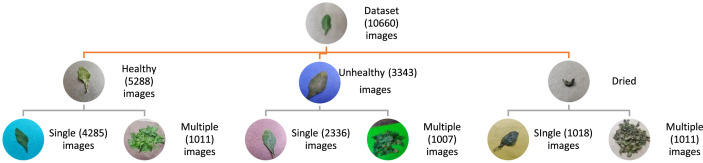
*Plumbago Zeylanica* (*Chitrak*) leaf image dataset folder structure and image counts.

The dataset contains 10,660 high-quality images of *Chitrak* leaves. The images are divided into three primary folders: Healthy (5288 images), Unhealthy (3343 images), and Dried (2029 images). Within each category, there is a division between “Single” and “Multiple” images. In the case of “Single” images, they are further classified into “Front” and “Back” perspectives. This structured organization makes it easy to navigate and retrieve specific leaf images. Table 1 shows *Chitrak* leaf image dataset categories and counts per category. Fig. 1 illustrates *Chitrak* leaf image dataset folder structure and image counts. Table 2 shows exemplar images from the dataset.

**Table 1:** *Plumbago Zeylanica* (*Chitrak*) leaf image categories and counts.

Sr No	Categories	Number of images
1.	Unhealthy	3343
2.	Healthy	5288
3.	Dried	2029
Total		**10,660**

**Table 2:** Exemplar Images from the dataset.

## 4 Experimental Design, Materials and Methods

### 4.1 Experimental setup

**Fig. 2:**
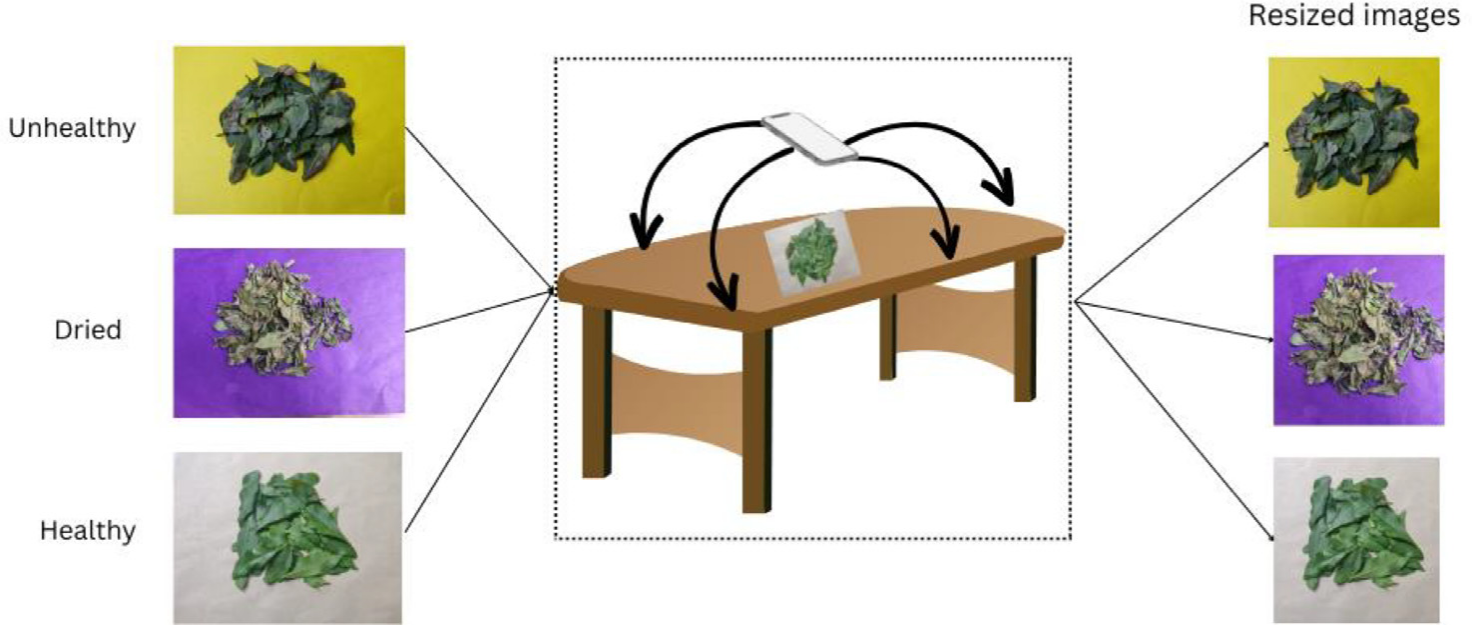
Experimental setup for *Chitrak* leaf photography dataset creation.

The experimental setup for creating the *Chitrak* leaf image dataset involved capturing high-resolution images of *Chitrak* leaves in their natural environment. *Chitrak* specimens are ubiquitously distributed across the Vishwakarma University campus. Leaves of the *Chitrak* plant were systematically gathered from the herbal garden of Vishwakarma University Pune (18°27′34.8″N 73°53′01.1″E) during the period spanning August 2023 to October 2023. A total of ten individual plants were selected for the purpose of leaf collection to construct the dataset. Photographs of all chosen plant leaves were captured in diverse backgrounds, angles, and lighting conditions. Fig. 2 illustrates experimental setup for *Chitrak* leaf images dataset creation.

### 4.2 Materials or specification of image acquisition system

The dataset creation involved compiling 10,660 high-quality images of *Chitrak* leaves, captured with a OnePlus 6 smartphone boasting a camera system comprising a 16-megapixel lens (f/1.7, 1.22-micron) and a 20-megapixel lens (f/1.7, 1.0-micron) with phase detection autofocus technology.

### 4.3 Pre-processing method

**Fig. 3:**
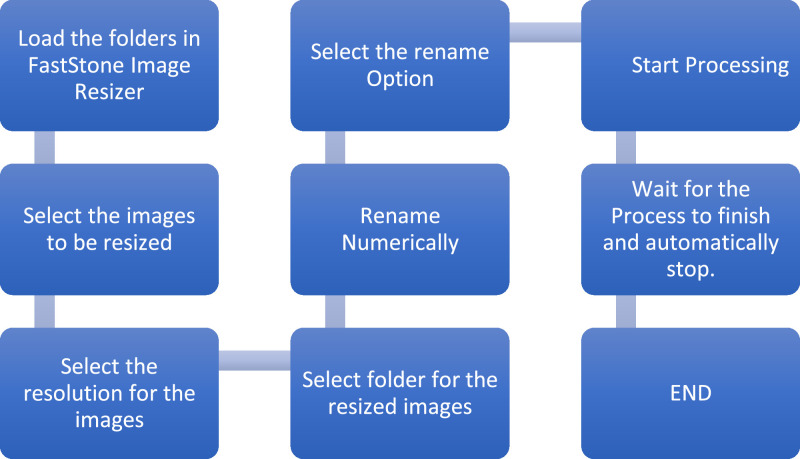
Preprocessing steps in the dataset.

To standardize the dataset, the images were uniformly resized to 1024 × 768 pixels using FastStone Photo Resizer. Subsequently, a systematic renaming process assigned sequential numerical identifiers to each image, ranging from 1 to 10,660, enhancing data management. Fig. 3 shows steps used for preprocessing on the dataset.

The images, sourced from various angles and backgrounds, comprehensively represented diverse leaf conditions. The Yogesh Suryawanshi (PhD in Botany), undertook the task of leaf classification. The classification criteria were based on visual attributes, where leaves exhibiting a clear green coloration without any discernible spots were categorized as “Healthy.” Conversely, leaves displaying visual spots, such as a whitish layer (Image 8328) or a yellow or grey coloration (Image 8568), were designated as “Unhealthy.” Dried leaves were gathered from their natural environment, and an analysis of moisture content was conducted. Leaves with a moisture content of less than 10% were categorized as “Dried” [10].

The dataset's structure involved a well-organized folder hierarchy, further segregating the data into “Single” and “Multiple” image subsets within each primary class. Within the “Single” images, categorization extended to distinguishing “Front” and “Back” perspectives. This rigorous approach to data collection, processing, and organization ensures both diversity and accessibility, catering to a wide range of research and analytical applications.

### 4.4 Demonstrating the significance of the dataset

In the domain of machine learning datasets, numerous significant contributions have arisen in recent times [2], [3], [4], [5], [6], [7], [8], addressing various machine learning applications. To underscore the significance of our *Plumbago Zeylanica* (Chitrak) dataset [1], we conducted experiments employing established pre-trained models such as InceptionV3, Xception, and EfficientNetB0. Our objective was to assess the dataset's potential in enhancing the performance of machine learning models, particularly in the tasks of accurately classifying *Chitrak* leaves and detecting signs of leaf diseases.

Initially, we evaluated these pre-trained models using our dataset as a benchmark without any modifications. Subsequently, we enhanced the performance of these models by fine-tuning them with our *Chitrak* dataset. In the implementation of our machine learning algorithm, a strategic division of the dataset was employed to optimize training, testing, and validation processes. Specifically, 75% of the dataset images (7995) were allocated for training purposes, while the remaining 25% (2665 images) were reserved for both testing and validation. This division facilitated a comprehensive evaluation of the algorithm's performance, as reflected in the confusion matrix presented in Table 4, which specifically pertains to this 25% subset [11]. This approach not only ensured a robust assessment of the algorithm's generalization capabilities but also aligns with best practices in machine learning experimentation. The results were quite compelling. Fine-tuning with our dataset led to a significant increase in accuracy, particularly in the precise classification of *Chitrak* leaves and the detection of disease symptoms. Table 3 provides a comparison of accuracy scores for pretrained machine learning models on the *Chitrak* dataset before and after fine-tuning, while Table 4 illustrates the confusion matrices. In summary, our *Plumbago Zeylanica* (*Chitrak*) dataset plays a pivotal role in enhancing the performance of machine learning models such as InceptionV3, Xception, and EfficientNetB0. Serving as a robust resource for training and fine-tuning, our dataset becomes an essential tool in the development of more reliable models, ultimately contributing to the betterment of *Chitrak* cultivation and plant health management.

**Table 3:** A comparison of accuracy scores for pretrained machine learning models on the *Plumbago Zeylanica* (*Chitrak*) dataset before and after fine-tuning.

Machine learning model	Accuracy (Before training on our dataset)	Accuracy (After training on our dataset)
InceptionV3	30.00%	94.60%
Xception	34.24%	95.50%
EfficientNetB0	35.67%	96.76%

**Table 4:** Confusion matrices on pretrained machine learning models on the *Plumbago Zeylanica* (*Chitrak*) dataset before and after training with our dataset.

## Limitations

The *Plumbago Zeylanica* (Chitrak) leaf image dataset lacks specific disease categorization.

## Ethics Statement

Our study does not involve studies with animals or humans. Therefore, we confirm that our research strictly adheres to the guidelines for authors provided by Data in Brief terms of ethical considerations.

## CRediT Author Statement

**Kailas Patil:** Conceptualization, Supervision, Writing – review & editing. **Yogesh Suryawanshi:** Conceptualization, Data Curation, Writing – review & editing. **Amit Dhoka:** Methodology. **Prawit Chumchu:** Writing – review & editing.

## Data Availability

Plumbago Zeylanica (Chitrak) Leaf Image Dataset (Original data) (Mendeley Data).
